# Relationship between immunohistochemical markers and clinical pathological variables in clear cell renal cell carcinoma

**DOI:** 10.3389/fonc.2025.1580024

**Published:** 2025-08-04

**Authors:** Pengshuai Liu, Junli Wei, Shubo Chen

**Affiliations:** ^1^ Department of Surgery, Graduate School of Chengde Medical College, Chengde, Hebei, China; ^2^ Department of Urology, Xingtai People’s Hospital, Xingtai, Hebei, China

**Keywords:** clear cell renal cell carcinoma, CD10, Vimentin, Ki-67, biomarker

## Abstract

**Objective:**

To explore the factors associated with the immunohistochemical results in clear cell renal cell carcinoma (ccRCC).

**Methods:**

This retrospective, single-center observational study included patients with pathologically confirmed ccRCC who underwent nephrectomy at Xingtai People’s Hospital between January 2023 and October 2024. Logistic and linear regression was used to evaluate predictors of ccRCC features, with adjustments for demographic and clinical factors.

**Result:**

Among the 50 patients (median age, 66 years; 64% male), high Vimentin expression was significantly associated with vascular invasion (adjusted OR, 2.90; 95% CI, 1.05–7.62; P = 0.031), lymph node metastasis (OR, 2.62; 95% CI, 1.03–8.12; P = 0.042), distant metastasis (OR, 3.10; 95% CI, 1.01–12.54; P = 0.048), and larger tumor size (P = 0.004). Ki−67 expression varied significantly by alcohol consumption (P = 0.024), perineural invasion (P = 0.038), and was positively correlated with serum creatinine (P = 0.017). CD10 expression was inversely correlated with bilirubin levels (P = 0.021) but not associated with invasive or metastatic features.

**Conclusion:**

Vimentin expression was strongly associated with markers of tumor invasiveness and may serve as a practical prognostic biomarker in ccRCC. Ki-67 may reflect proliferative activity and systemic burden. CD10 remains diagnostically useful but lacks prognostic value. Incorporating these IHC markers into routine pathology assessment may enhance risk stratification and inform individualized management of ccRCC with more exploration and validation.

## 1 Introduction

Renal cell carcinoma (RCC) is a clinically heterogeneous malignancy that accounts for approximately 2-3% of all adult cancers worldwide (1–4). Its incidence has steadily increased in recent decades due to improvements in imaging examinations and changes in environmental and metabolic risk factors such as obesity, hypertension, and smoking. Among its histological subtypes, clear cell renal cell carcinoma (ccRCC) is the most common, accounting for nearly 75% of all RCC cases (2, 5, 6).

Despite advances in surgical and systemic therapies, the treatment of ccRCC remains challenging due to its unpredictable clinical behavior, the potential for late recurrence, and resistance to conventional treatments (7). Accurate risk stratification is essential to guide treatment decisions, but conventional parameters such as tumor stage, grade, and morphology often lack adequate prognostic precision. Immunohistochemical staining (IHC) have emerged as a practical and cost-effective tool to complement histopathological assessment, especially in settings with limited molecular testing. Some markers are commonly overexpressed in ccRCC and may function as specific indicators for the diagnosis and differential diagnosis of this condition (8–10). For example, CD10, a cell surface metalloproteinase, is normally expressed in the proximal tubular epithelium and is often positive in ccRCC. It is associated with tumor progression and is used to diagnostically distinguish ccRCC from other renal tumours (11, 12). vimentin is associated with epithelial-mesenchymal transition and tumor invasiveness (13–18)., and Ki-67 can be used as a marker of cell proliferation (19–21).

Currently, it remains unclear how these markers work in combination and whether their collective expression patterns can more accurately reflect tumor biology and predict clinically relevant features. Limited studies have evaluated the relationship between the combined utility of these markers and pathological aggressiveness, stage progression, or risk of metastasis. This study aimed to describe the immunohistochemical expression patterns of CD10, Vimentin, and Ki-67 in ccRCC and investigate their associations with key clinicopathological features.

## 2 Methods

### 2.1 Study design and methods

#### 2.1.1 Study subjects

This single-center, retrospective, observational study was done at Xingtai People’s Hospital. We collected data from patients who were newly diagnosed with ccRCC at the Department of Urology from January 2023 to October 2024.

Patients were eligible for inclusion if they were 18 years of age or older, had a histopathological diagnosis of ccRCC, and underwent partial or radical nephrectomy during the study period. Only patients who had not received prior oncologic treatment, including systemic therapy, radiation, or surgery for renal malignancy, were included. Exclusion criteria consisted of incomplete clinical records, inadequate tumor tissue for IHC evaluation, concurrent malignancy, or prior history of renal cancer. A total of 56 patients were initially identified, of whom 6 were excluded due to missing data, resulting in a final study cohort of 50 patients.

Demographic and clinical data were collected including age, sex, height, weight, body mass index (BMI), preoperative liver and kidney function, tumor size (in centimeters), tumor stage [TNM classification (22)], histologic grade [according to WHO criteria (23)], and the presence or absence of vascular invasion, capsule invasion, nerve invasion, lymph node metastasis, and distant metastasis.

### 2.2 Pathological examination methods

The expression of immunohistochemical markers, including Vimentin, Ki-67, and CD10, was collected. In this study, specimens were preserved using 10% neutral formalin, subsequently subjected to standard dehydration processes, embedded in paraffin, and sectioned to a thickness of 4 μm. The antibodies employed for analysis included Vimentin, Ki-67, and CD10. The criteria for interpreting immunohistochemical staining are as follows:

CD10 (24) and Vimentin (25) expression were assessed based on cytoplasmic staining. A semi-quantitative scoring system was used, incorporating both staining intensity (0 = no staining, 1 = light yellow, 2 = brown, 3 = dark brown) and the percentage of positively stained tumor cells (0: ≤10%, 1: 11–25%, 2: 26–50%, 3: 51–75%, 4: >75%). A composite score was generated by multiplying intensity and proportion scores, with a final score ≤3 indicating low expression and ≥4 indicating high expression.

The criteria for determining Ki-67 positivity are predicated on the observation of nuclear staining exhibiting a brown-yellow hue. At a magnification of 400x, ten representative fields are selected for analysis, within which 100 tumor cells are enumerated per field to ascertain the percentage of cells exhibiting positive staining (26). A lack of stained tumor cells is classified as negative. A staining rate of less than 10% is categorized as weakly positive, between 10% and 30% as positive, and greater than 30% as strongly positive. All clinical and pathological data were reviewed and verified by trained research personnel.

### 2.3 Statistical methods

Categorical variables were reported as proportions or percentages, whereas continuous variables were expressed as median and IQR values. Differences in IHC marker expression across categorical clinicopathological features were analyzed using the chi-square (χ²) test or Fisher’s exact test, where appropriate. To visualize the relationship between immunohistochemical markers and clinical parameters, a correlation heatmap was constructed. Multivariate logistic regression and linear regression models were constructed to evaluate the independent association between IHC markers and key clinical or pathological variables, adjusting for potential confounders. Model 1 is a crude model which is unadjusted. Model 2 is adjusted for age and gender. Model 3 is further adjusted for clinical variables, including BMI, hypertension, coronary heart disease, diabetes, smoking, and drinking. Crude and adjusted odds ratios (ORs) and beta with 95% confidence intervals (CIs) were reported. P values were from 2-sided tests, and results were deemed statistically significant at P < .05. Statistical analyses were performed using SPSS version 27.0.

## 3 Result

**Figure 1:**
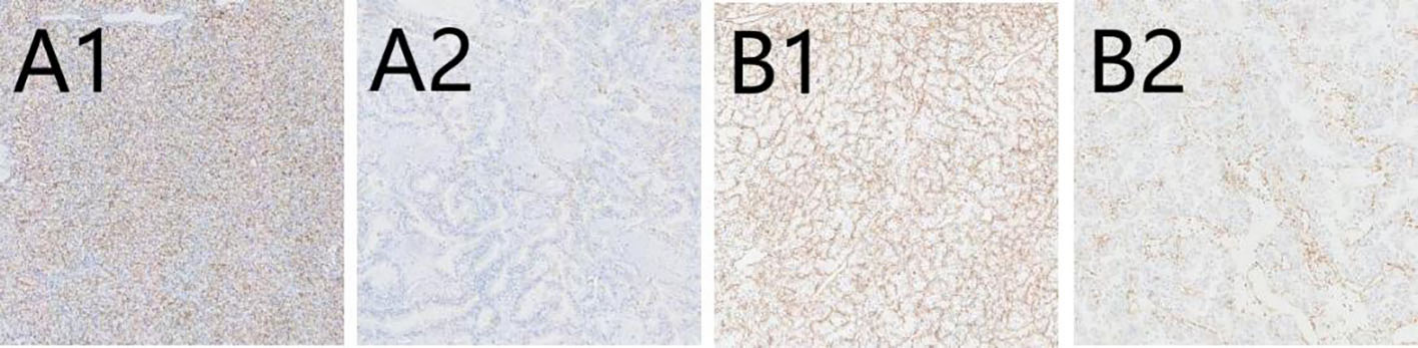
The immunohistochemical expression of CD10 and Vimentin in CCRCC. **(A1)** High expression of CD10 (×200); **(A2)** Low expression of CD10 (×200); **(B1)** High expression of Vimentin (×200); **(B2)** Low expression of Vimentin (×200).

**Figure 2:**
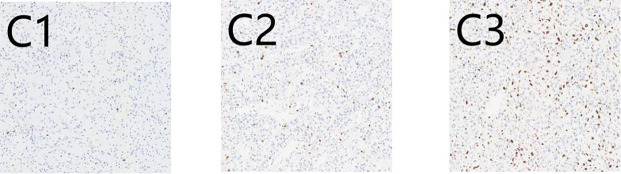
Immunohistochemical expression of Ki67 in CCRCC.C1: **(C1)** Weakly positive (×400); **(C2)** Positive (×400); **(C3)** Strongly positive (×400).

There are 50 patients diagnosed with ccRCC included in our study. The majority of those is male (64%; 36% were female), and median (IQR) age is 66 (56, 72) years. The baseline characteristics are described in **Table 1**. IHC staining patterns of the three evaluated markers, CD10, Vimentin, and Ki-67, are presented in **Figures 1** and 2. Specifically, **Figures 1A1, A2** illustrate high and low CD10 expression, respectively, while **Figures 1B1, B2** demonstrate high and low Vimentin expression, each at 200× magnification. **Figure 2** shows representative staining patterns of Ki-67 at 400× magnification, with weakly positive (**Figure 2C1**), positive (**Figure 2C2**), and strongly positive expression levels (**Figure 2C3**) indicating variable nuclear staining intensity.

**Table 1:** Baseline table.

Characteristic	N = 50
Gender, n (%)	
Female	29 (58%)
Male	21 (42%)
Age, Median (IQR)	66 (56, 72)
Body Mass Index (kg/m²), Median (IQR)	23.3 (21.6, 25.1)
Tumor Size (cm), Median (IQR)	4.53 (3.90, 5.08)
Alanine Aminotransferase (U/L), Median (IQR)	25 (18, 31)
Aspartate Aminotransferase (U/L), Median (IQR)	24 (20, 32)
Albumin (g/L), Median (IQR)	39.4 (36.4, 42.7)
Total Bilirubin (µmol/L), Median (IQR)	12.4 (10.0, 16.1)
Direct Bilirubin (µmol/L), Median (IQR)	8.1 (5.0, 11.9)
Alkaline Phosphatase (U/L), Median (IQR)	85 (70, 92)
Gamma-Glutamyl Transferase (U/L), Median (IQR)	34 (28, 42)
Serum Creatinine (µmol/L), Median (IQR)	99 (92, 108)
Ki-67 Immunohistochemical Expression, n (%)	
Positive	20 (40%)
Strong Positive	7 (14%)
Weak Positive	23 (46%)
Vimentin Immunohistochemical Expression, n (%)	
High Expression	22 (44%)
Low Expression	28 (56%)
CD10 Immunohistochemical Expression, n (%)	
High Expression	44 (88%)
Low Expression	6 (12%)
History of Hypertension, n (%)	33 (66%)
History of Coronary Heart Disease, n (%)	9 (18%)
History of Diabetes Mellitus, n (%)	12 (24%)
Smoking History, n (%)	15 (30%)
Alcohol Consumption History, n (%)	18 (36%)
Vascular Invasion Present, n (%)	30 (60%)
Capsular (Membrane) Violation Present, n (%)	21 (42%)
Perineural Infiltration Present, n (%)	48 (96%)
Lymph Node Infiltration Present, n (%)	30 (60%)
Distant Tumor Metastasis Present, n (%)	38 (76%)
Tumar size (cm), Median (IQR)	5.20 (4.40- 5.91)
Tumor Stage, n (%)	
Phase I	13 (26%)
Phase II	5 (10%)
Phase III	26 (52%)
Phase IV	6 (12%)
Tumor Grade, n (%)	
Grade I	21 (42%)
Grade II	28 (56%)
Grade III	1 (2%)

**Figure 3:**
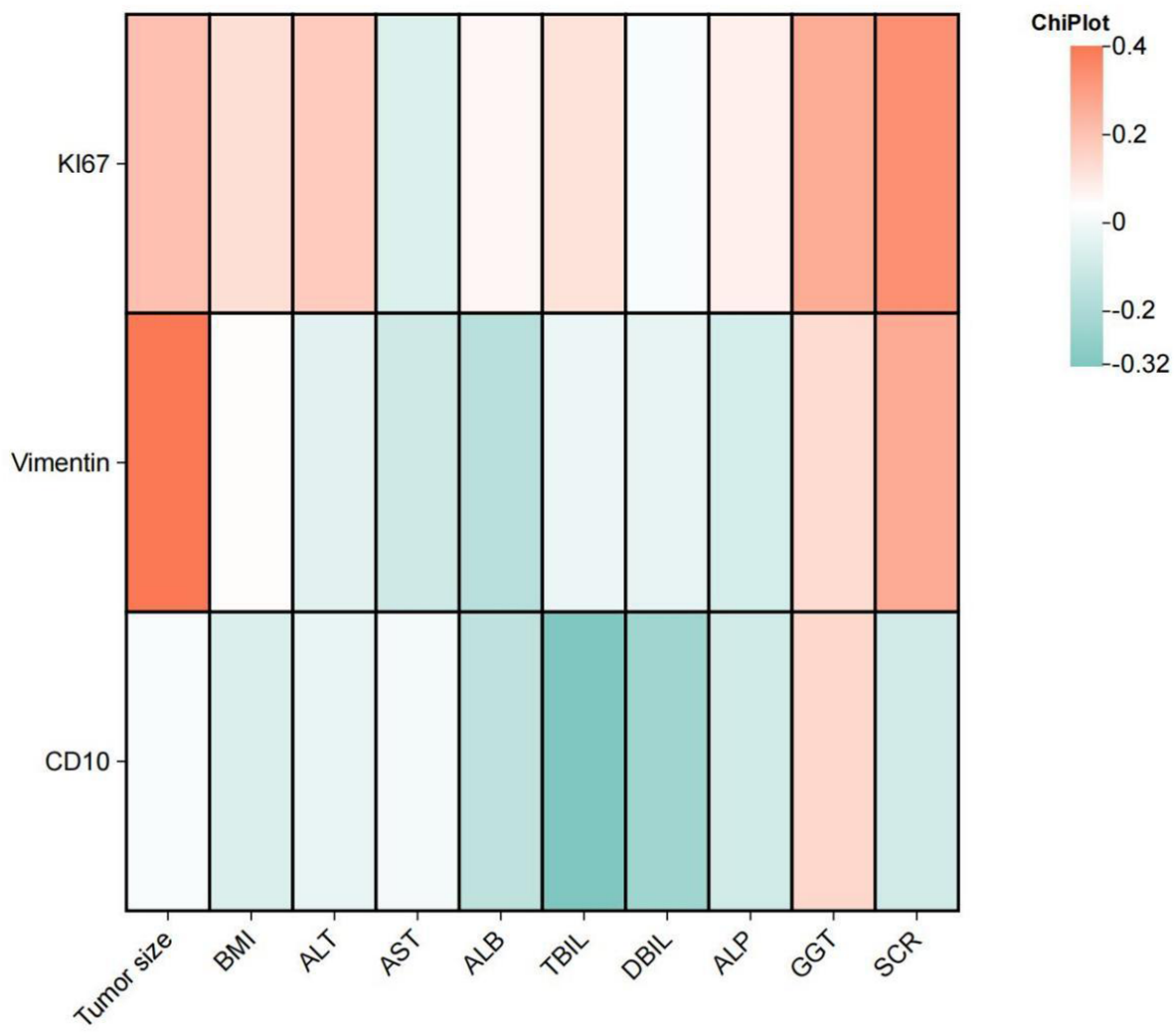
The heatmap illustrates the correlation between Vimentin, Ki-67, and CD10 with tumor size, BMI, and liver and kidney function in patients with clear cell renal cell carcinoma. ALT, Alanine Aminotransferase; AST, Aspartate Aminotransferase; ALB, Albumin; TBIL, Total Bilirubin; DBIL, Direct Bilirubin; ALP, Alkaline Phosphatase; GGT, Gamma-Glutamyl Transferase; SCR, Serum Creatinine.

A significant difference in Ki-67 expression is observed between patients with and without a history of alcohol consumption (P = .024) as well as between those with and without perineural invasion (P = .038). More information comparing the difference across the IHC marker is shown in **Supplementary Tables S1**, **S2**. A positive correlation between Ki-67 expression and serum creatinine (SCR) levels (r = 0.337, P = 0.017), between Vimentin expression and tumor size (r = 0.400, P = 0.04) is suggested. And CD10 expression exhibited a negative correlation with total bilirubin (TBIL) levels (r = -0.325, P = 0.021). Comprehensive details of the correlation analysis are provided in **Figure 3**.

After adjusting for age and sex, high expression of Vimentin was significantly associated with vascular invasion (OR, 2.90; 95% CI, 1.05–7.62; P = .031), lymph node involvement (OR, 2.62; 95% CI, 1.03–8.12; P = .042), and distant metastasis (OR, 3.10; 95% CI, 1.01–12.54; P = .048), as detailed in **Table 2**. No statistically significant associations were observed for Ki-67 or CD10 positive expression with any of the ccRCC-related outcomes. And Vimentin high-level expression is observed significant association with the tumor size (beta, 1.02 95% CI, 0.41–1.63; P =0.002). All regression result is reported in **Supplementary Tables S3**, **S4**. 

**Table 2:** Association between IHC predictors and ccRCC.

Outcome	IHC predictors	Model	OR	95%CI
Lymph Node	Ki67_High	Model2	0.88	(0.16, 5.28)
Lymph Node	Vimentin_High	Model2	2.46*	(1.03, 8.12)
Lymph Node	CD10_High	Model2	0.95	(0.11, 6.41)
Vascular Invasion	Ki67_High	Model2	0.45	(0.08, 2.39)
Vascular Invasion	Vimentin_High	Model2	2.90*	(1.05, 7.62)
Vascular Invasion	CD10_High	Model2	0.80	(0.1, 5.04)
Metastasis	Ki67_High	Model2	0.81	(0.13, 6.66)
Metastasis	Vimentin_High	Model2	2.95*	(1.01,12.54
Metastasis	CD10_High	Model2	0.30	(0.01, 2.55)

*p<0.05.

Model 2: adjusted for age and gender.

## 4 Discussion

This study investigated the immunohistochemical expression patterns of Vimentin, Ki-67, and CD10 with ccRCC and explored their associations with key clinical and pathological features. Vimentin expression was significantly associated with pathological indicators, including vascular invasion, lymph node involvement, and distant metastasis. Ki-67 expression showed significant differences based on alcohol consumption and the presence of perineural invasion, and correlated positively with serum creatinine levels. CD10 expression was not significantly associated with major clinical outcomes, though it showed an inverse correlation with total bilirubin. These findings suggest that Vimentin and Ki-67, in particular, may offer additional prognostic value beyond conventional histopathological parameters in ccRCC (10, 27).

Our findings that Vimentin expression tracks with tumor invasiveness and metastatic spread demonstrate its role in epithelial–mesenchymal transition (EMT), a critical process in cancer progression (28). Vimentin has long been used diagnostically in RCC, but its role as a marker of biologic aggressiveness is increasingly recognized. Prior studies have reported that Vimentin overexpression correlates with poor survival in RCC, largely through its role in cytoskeletal reorganization, cell motility, and matrix degradation (29–32). Mechanistic studies also show that tumor necrosis factor-alpha (TNF−α) can upregulate Vimentin and downregulate E-cadherin, thereby promoting matrix metalloproteinase-9 (MMP9) activation and invasion (33–35). The associations we observed with vascular invasion and metastasis strengthen this functional link and suggest that Vimentin expression in routine pathology could serve as a surrogate marker of EMT activation in ccRCC.

From a clinical perspective, the consistent associations observed for Vimentin suggest that its expression may serve as a practical surrogate for tumor aggressiveness. In the absence of genomic profiling, high Vimentin could help flag patients at elevated risk of progression who might benefit from more intensive postoperative surveillance or earlier systemic therapy. Given its routine availability in pathology labs and straightforward scoring, Vimentin offers a low-cost, scalable addition to prognostic frameworks, particularly in resource-constrained settings.

In contrast, CD10 did not demonstrate strong associations with pathologic aggressiveness in our analysis. While its diagnostic value is well supported in the differentiation of ccRCC from other subtypes, its prognostic significance remains controversial (12, 36). The inverse correlation between CD10 and bilirubin levels may reflect an association with systemic liver function or tumor-related metabolic shifts, as noted in prior studies on metastatic liver disease, but this requires further exploration (37, 38).

Ki−67 expression, though not statistically associated with metastasis in our study, was significantly different across categories of drinking and perineural invasion and showed a positive correlation with serum creatinine. These findings are consistent with Ki−67’s role as a proliferation marker that reflects both tumor cell kinetics and, potentially, systemic host-tumor interactions. Previous studies have found Ki−67 to be associated with higher tumor grade, increased mitotic activity, and shorter recurrence-free survival in RCC (39–41). The correlation we observed with serum creatinine may indicate a link between tumor activity and renal functional decline, which could reflect tumor burden or cachexia-related sarcopenia (42, 43). The variation by alcohol use also aligns with evidence showing that alcohol may influence tumor proliferation, including via inflammatory or metabolic pathways (44, 45). This raises the potential importance of managing alcohol consumption as part of daily supportive care.

Several limitations should be acknowledged. First, the single-center setting inherently introduces the potential for selection bias and constrains the generalizability of the results to broader, more diverse populations. Second, the modest sample size may limit the statistical power to detect subtle or complex associations, particularly in multivariable models and subgroup analyses. Third, the absence of longitudinal follow-up data precludes assessment of survival outcomes and limits the ability to draw prognostic inferences. Finally, while multivariate logistic regression was employed to adjust for key clinicopathological covariates, residual confounding from unmeasured biological or molecular factors cannot be fully assessed.

This study demonstrates that immunohistochemical markers, particularly vimentin and Ki-67, can provide clinically meaningful insights into the biological behavior of clear cell renal cell carcinoma. Vimentin expression was independently associated with tumor invasive and metastatic features, supporting its potential role as a prognostic marker. Ki-67 correlated with systemic indices and tumor-related factors, suggesting its utility in assessing proliferative activity and tumor burden. Although CD10 retained diagnostic relevance, it did not demonstrate prognostic value in our cohort. In conclusion, this study calls for further prospective validation in larger, multi-institutional cohorts in the future to confirm its prognostic utility and evaluate its role in treatment decisions.

## Data Availability

The raw data supporting the conclusions of this article will be made available by the authors, without undue reservation.
