# Supplementary material for: Relationship between immunohistochemical markers and clinical pathological variables in clear cell renal cell carcinoma
Source: Front Oncol. 2025 Aug 4;15:1580024. doi: 10.3389/fonc.2025.1580024 (PMC12358267; doi:10.3389/fonc.2025.1580024)
Supplement: Supplementary file 1 [file Table1.docx]

Table S1: Difference across CD10 and Vimentin expression

|  | CD10 |  |  | Vimentin |  |  |
| --- | --- | --- | --- | --- | --- | --- |
|  | High expression | Low expression | P | High expression | Low expression | P |
| Gender |  |  |  |  |  |  |
| Female | 16 | 2 | 0.342 | 10 | 8 | 0.63 |
| Male | 25 | 7 |  | 20 | 12 |  |
| Hypertension |  |  |  |  |  |  |
| Yes | 16 | 3 | 0.75 | 9 | 10 | 0.153 |
| No | 25 | 6 |  | 21 | 10 |  |
| Coronary heart disease |  |  |  |  |  |  |
| Yes | 4 | 1 | 0.902 | 3 | 2 | 1 |
| No | 37 | 8 |  | 27 | 18 |  |
| Diabetes |  |  |  |  |  |  |
| Yes | 5 | 1 | 0.928 | 3 | 3 | 0.594 |
| No | 36 | 8 |  | 27 | 17 |  |
| Smoke |  |  |  |  |  |  |
| Yes | 11 | 2 | 0.775 | 6 | 7 | 0.236 |
| No | 30 | 7 |  | 24 | 13 |  |
| Drinking |  |  |  |  |  |  |
| Yes | 7 | 2 | 0.716 | 6 | 3 | 0.652 |
| No | 34 | 7 |  | 24 | 17 |  |
| Vasculature infiltration |  |  |  |  |  |  |
| Yes | 34 | 8 | 0.659 | 28 | 14 | 0.027 |
| No | 7 | 1 |  | 2 | 6 |  |
| Membrane violation |  |  |  |  |  |  |
| Yes | 11 | 4 | 0.296 | 11 | 4 | 0.208 |
| No | 30 | 5 |  | 19 | 16 |  |
| Tumor metastasis |  |  |  |  |  |  |
| Yes | 38 | 9 | 0.403 | 29 | 18 | 0.331 |
| No | 3 | 0 |  | 1 | 2 |  |
| Perineural infiltration |  |  |  |  |  |  |
| Yes | 39 | 9 | 0.499 | 29 | 19 | 0.768 |
| No | 2 | 0 |  | 1 | 1 |  |
| Lymph node infiltration |  |  |  |  |  |  |
| Yes | 39 | 8 | 0.476 | 28 | 19 | 0.08 |
| No | 2 | 1 |  | 2 | 1 |  |
| Tumor stage |  |  |  |  |  |  |
| Phase I | 8 | 1 | 0.46 | 7 | 2 | 0.541 |
| Phase II | 3 | 2 |  | 3 | 2 |  |
| Phase III | 27 | 6 |  | 19 | 14 |  |
| Phase IV | 3 | 0 |  | 1 | 2 |  |

Table S2: Difference across Ki-67 expression

|  | Ki-67 |  |  |
| --- | --- | --- | --- |
|  | High expression | Low expression | P |
| Gender |  |  |  |
| Female | 12 | 5 | 0.197 |
| Male | 13 | 17 |  |
| Hypertension |  |  |  |
| Yes | 11 | 7 | 0.628 |
| No | 14 | 15 |  |
| Coronary heart disease |  |  |  |
| Yes | 3 | 2 | 0.793 |
| No | 22 | 20 |  |
| Diabetes |  |  |  |
| Yes | 3 | 2 | 0.48 |
| No | 22 | 20 |  |
| Smoke |  |  |  |
| Yes | 5 | 6 | 0.216 |
| No | 20 | 16 |  |
| Drinking |  |  |  |
| Yes | 2 | 5 | 0.033 |
| No | 23 | 17 |  |
| Vasculature infiltration |  |  |  |
| Yes | 22 | 18 | 0.659 |
| No | 3 | 4 |  |
| Membrane violation |  |  |  |
| Yes | 7 | 7 | 0.296 |
| No | 18 | 15 |  |
| Tumor metastasis |  |  |  |
| Yes | 23 | 21 | 0.403 |
| No | 2 | 1 |  |
| Perineural infiltration |  |  |  |
| Yes | 25 | 21 | 0.02 |
| No | 0 | 1 |  |
| Lymph node infiltration |  |  |  |
| Yes | 24 | 20 | 0.69 |
| No | 1 | 2 |  |
| Tumor stage |  |  |  |
| Phase I | 6 | 2 | 0.192 |
| Phase II | 0 | 5 |  |
| Phase III | 18 | 14 |  |
| Phase IV | 2 | 1 |  |

Table S3: All regression result between tumour outcome and IHC markers

| Outcome | IHC | Model | OR | CI |
| --- | --- | --- | --- | --- |
| Lymph Node | Ki67_High | Model1 | 0.87 | (0.17, 4.89) |
| Lymph Node | Ki67_High | Model2 | 0.88 | (0.16, 5.28) |
| Lymph Node | Ki67_High | Model3 | 0.44 | (0.05, 3.62) |
| Lymph Node | Vimentin_High | Model1 | 1.86 | (0.59, 6.18) |
| Lymph Node | Vimentin_High | Model2 | 2.46* | (1.03, 8.12) |
| Lymph Node | Vimentin_High | Model3 | 1.51 | (0.3, 7.91) |
| Lymph Node | CD10_High | Model1 | 0.72 | (0.09, 4.12) |
| Lymph Node | CD10_High | Model2 | 0.95 | (0.11, 6.41) |
| Lymph Node | CD10_High | Model3 | 0.7 | (0.06, 7.6) |
| Vascular Invasion | Ki67_High | Model1 | 0.44 | (0.08, 2.26) |
| Vascular Invasion | Ki67_High | Model2 | 0.45 | (0.08, 2.39) |
| Vascular Invasion | Ki67_High | Model3 | 0.62 | (0.09, 3.81) |
| Vascular Invasion | Vimentin_High | Model1 | 1.31 | (0.42, 4.23) |
| Vascular Invasion | Vimentin_High | Model2 | 2.9* | (1.05, 7.62) |
| Vascular Invasion | Vimentin_High | Model3 | 1.07 | (0.24, 4.94) |
| Vascular Invasion | CD10_High | Model1 | 0.72 | (0.09, 4.12) |
| Vascular Invasion | CD10_High | Model2 | 0.8 | (0.1, 5.04) |
| Vascular Invasion | CD10_High | Model3 | 1.14 | (0.11, 11.62) |
| Metastasis | Ki67_High | Model1 | 0.76 | (0.14, 5.85) |
| Metastasis | Ki67_High | Model2 | 0.81 | (0.13, 6.66) |
| Metastasis | Ki67_High | Model3 | 1.12 | (0.15, 11.86) |
| Metastasis | Vimentin_High | Model1 | 3 | (0.76, 15.13) |
| Metastasis | Vimentin_High | Model2 | 2.95* | (1.01,12.54 |
| Metastasis | Vimentin_High | Model3 | 2.36 | (0.43, 15.86) |
| Metastasis | CD10_High | Model1 | 0.6 | (0.03, 4.28) |
| Metastasis | CD10_High | Model2 | 0.3 | (0.01, 2.55) |
| Metastasis | CD10_High | Model3 | 0.34 | (0.01, 4.57) |

Model 1: unadjusted model

Model 2: adjusted for age and gender

Model 3: adjusted for age, gender, BMI, hypertension, coronary heart disease, diabetes, smoking, and drinking.

Table S4: Association between IHC markers expression and tumour size

| Variable | Model | Effect size | 95%CI |
| --- | --- | --- | --- |
| Ki67_High | Model 1 | 0.38 | (-0.20, 0.96) |
|  | Model 2 | 0.35 | (-0.28, 0.98) |
|  | Model 3 | 0.32 | (-0.36, 0.99) |
| Vimentin_High | Model 1 | 1.05* | (0.45, 1.66) |
|  | Model 2 | 1.02* | (0.41, 1.63) |
|  | Model 3 | 0.98* | (0.38, 1.59) |
| CD10_High | Model 1 | 0.12 | (-0.85, 1.09) |
|  | Model 2 | 0.10 | (-0.88, 1.09) |
|  | Model 3 | 0.08 | (-0.95, 1.12) |

Model 1: unadjusted model

Model 2: adjusted for age and gender

Model 3: adjusted for age, gender, BMI, hypertension, coronary heart disease, diabetes, smoking, and drinking.
